# Fast-Response Electrostatic Actuator Based on Nano-Gap

**DOI:** 10.3390/mi8030078

**Published:** 2017-03-03

**Authors:** Edward Kostsov, Alexei Sokolov

**Affiliations:** Lab Thin Ferroelectric Films Structure, Institute of Automation and Electrometry, Russian Academy of Science, Siberian Branch, Koptug Prosp.1, Novosibirsk 630090, Russia; sokolovaa@iae.sbras.ru

**Keywords:** electrostatic actuator, large force, nanometer gap, ferroelectric, fast driver

## Abstract

The possibility of constructing new high-performance electrostatic fast actuators based on energy transformation in nanometer gaps is considered. The construction and the properties of the operation of such devices as well as their typical parameters are described. The drives are based on ferroelectrics with high values of dielectric permittivity (above 1000). They can be constructed using microelectronic technology. It is demonstrated that the actuators are capable of maintaining forces with a specific density up to 10^6^ N/m^2^ and up to 100–1000 N in real devices for 10–100 µs. Experimental research results of such actuators are presented.

## 1. Introduction

A number of applications need actuators to be of small size, to develop large forces on a scale of less than a millisecond—for example, devices for fuel injection—to have “needleless” syringes and so on. To date, traditional solenoidal actuators have exhausted their potential. Over the last few years, actuators based on piezoelectric ceramics (PZT) have been widely used [1,2,3,4,5,6]. In some applications, an actuator has to be able to produce high mechanical force (100–500 N) in short times (100 µs or less). Piezoelectric actuators based on the reverse piezoelectric effect are electromechanical energy conversion devices that can achieve these parameters. For piezoelectric actuators, in order to achieve sufficient stroke values (10–50 µm), 100–500 ceramic plates, each 25–200 µm thick, have to be assembled into a single stack [1,4]. The packet length is 8 × 10^−2^–1 × 10^−1^ m, and its volume is 5 × 10^−6^–1 × 10^−5^ m^3^. Even though piezoelectric actuators are mass produced, their parameters are still not optimal. Among the main drawbacks of the piezoelectric actuators are complex production technology and relatively low reliability due to the multilayer structure of the piezoelectric drive and its fragility.

Microelectromechanical (MEMS) actuators [7,8,9,10,11] can potentially address these problems, because they can be manufactured more easily, and are more reliable and fast-acting than the piezoelectric ones. Electrostatic actuators have the most potential among them. Classic electrostatic MEMS actuators (EA) achieve only small forces because the electric field (*E*) in the gap is not high enough. The value of *E* determines the basic parameters of EA: thrust, power, response time. However, even though modern MEMS that are based on 2–3 µm air gaps have a high enough operating speed, they do not have sufficient energy density (it is less than 0.01 J/m^2^) and therefore cannot produce force that is more than 10 µN. They cannot achieve a sufficient stroke for the moving element of the construction; this stroke is limited to 3–5 µm.

In [10,11] a way to decrease the gap of EA, up to 200 nm, is described. Achieved actuator forces in this work do not exceed, as estimated, 0.01 N and actuator travel ranges are small, about a few µm, despite the complex structure.

In this article, we describe a principle to produce a fast-response, large-force electrostatic actuator. A new mechanism of electromechanical energy conversion that can be used for high energy density actuators capable of producing high mechanical forces in a short time is considered.

## 2. Operation Principle of the Fast-Response Electrostatic Actuator

Earlier, we proposed a new mechanism of electromechanical energy conversion [12,13,14,15,16] that can considerably improve the actuator energy density *A*_s_ up to 1–10 J/m^2^. This mechanism is based on the energy conversion in a 10–500 nm gap. The gap size depends on the applied voltage. We have also developed a mathematical model of the process. The model describes the main properties of such devices. Model predictions agree with the experimental results.

The high efficiency of the electromechanical energy conversion is due to the use of the dielectric thick layers (ferroelectric films) with high ε*/d* values of more than 10^8^–10^9^ m^−1^ (ε is the relative permittivity, *d* is the thickness of the ferroelectric layer). In this case, in the electrode–ferroelectric–nanogap–moving electrode (thin free metallic petal) structure, almost all the applied voltage drops in the nanogap, and thus the energy density of the electric field in the gap reaches 10^8^ J/m^3^ and the pressure reaches values of 3–10 MPa.

When the electrode gap *z* changes and the constant potential difference (*V*) is applied between the electrodes, the specific electrostatic force *F*, acting on the moving electrode of the capacitor, equals 0.5*V*^2^(d*C*_z_/d*z*). This is the force of attraction of the electrodes.

The specific capacity of a two-layer capacitor consisting of two dielectric layers connected in a series (first layer: dielectric with size *d* and dielectric permittivity ε, second layer: air gap with variable size *d*_z_) equals:

(1) C=Cz⋅CdCz+Cd=Cz(1+CzCd)=ε0(dz+d/ε)

Then

(2) $$F=\frac{V^{2}}{2}\cdot \frac{dC}{dz}=-\frac{V^{2}}{2}\cdot \frac{\varepsilon_{0}}{{\left(d_{z}+d/\varepsilon \right)}^{2}}$$

In general, the value of *F*, as can be easily shown (see, for example, [14,15]), may vary within rather wide limits, from 1 to 100 MPa, depending on the combination of parameters (*V*, *d*_z_, *d*, ε). In particular, for the parameters specified in the description of the experiment (*V* = 300 V, *d*_z_ = 1.7 × 10^−7^ m, *d*/ε = 2 × 10^−8^ m), the value *F* was 22 MPa.

A fixed plate (FP) consists of a silicon or sapphire substrate, an electrode and a ferroelectric thin layer. A gap *d*_e_ separates a moving plate (MP) and the FP. Thin petals of length *l* and width *b* are mounted on the MP. The MP moves relative to the FP along guides. 

**Figure 1:**
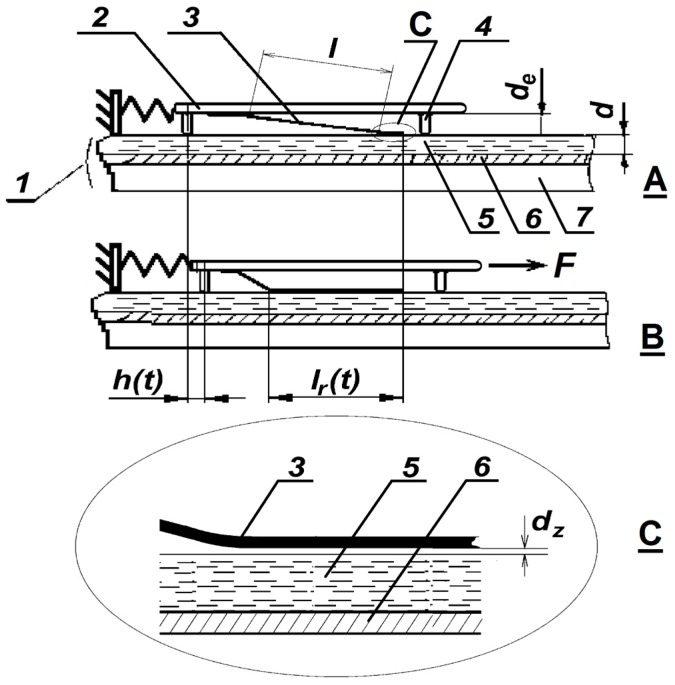
Schematic of electric to mechanical energy conversion during electrostatic rolling. 1—Fixed plate (FP), 2—Moving plate (MP), 3—Petal, 4—Guide ways, 5—Ferroelectric film, 6—Electrode, 7—Substrate. *l*—Free petal length before acting, *l*_r_(*t*)—Free petal length during acting, *h*(*t*)—Displacements, *d*_e_—The gap, *d*—Ferroelectric thickness, *d*_z_—Nanometer gap size, *F*—Produced force. (**A**) Start position, (**B**) position during acting, (**C**) zoom picture.

The motion of the MP relative to the FP consists of several stages:

(1) A voltage pulse with a magnitude *V* is applied between the petal in its initial state and the electrode. The petal’s tail is electrostatically attracted to the ferroelectric surface and forms a nanometer gap with size *d*_z_. The force acting on the petal is *F*_l_ = 0.5*V*^2^d*C*(*t*)/d*x*. The capacity *C*(*t*) of the electrode–thin ferroelectric layer–gap–petal (EFGP) structure equals ε_0_*S*(*t*)*/*(*d/*ε *+ d*_z_), where *S*(*t*) is the contact area of the two surfaces (rolling area *S*), and ε_0_ is the vacuum permittivity.
(2) The motion of the MP takes place as a bigger fraction of the petal rolls onto the ferroelectric surface, and the petal is bent and mechanically stretched, as shown in Figure 1С. Thus the electromechanical energy conversion takes place and the MP comes into state B. The rolling length *l*_r_(*t*), *S = bl*_r_(*t*) and *C*(*t*) grow during the voltage pulse, and the displacement *h*(*t*) of the MP increases. The *h*(*t*) and rolling speed depend on the mass *m* of the MP, the elasticity *k* of the spring, the applied voltage *V* and the friction coefficient η.
(3) The MP motion is preceded by a short process, when the petal is electrostatically pressed to the ferroelectric film, as shown in Figure 1C. The duration of the process depends on the applied voltage. It is about 1–5 µs in real experiments. The force of this initial attraction has to be large enough to prevent the sliding of the petal.

In [16] we introduced the concept of fast reversible "electronic glue". The specific force of the shift of the free metallic plate on ferroelectric film, the tangential force *F*_tg_, sufficient to remove these surfaces one from another, is determined by the rolling energy. It was experimentally determined as 3 × 10^5^–5 × 10^5^ N/J [16]. The knowledge of *F*_tg_ enables us to determine the minimum contact length, *l*_min_, when the MP movement starts, *l*_min_* > mg/AbF*_tg_.

The estimated value of *l*_min_ is 3−10 mm, and it is significantly less than the length of rolling *l*_r_(*t*).

A number of high-energetic-output micro-motor prototypes have been created using both ferroelectric films and thin antiferroelectric ceramics [12,13]. In these studies, the authors used the described energy conversion principle. The following parameters were used: *A*_s_ = *C*_s_*V*^2^/2 = 1–10 J/m^2^, pulse duration *t*_p_ = 20–400 µs, MP stroke 0.01–30 µm with a mass *m* of about 5 × 10^–3^−1 × 10^−1^ kg, voltage *V* = 20–100 V. A specific force up to 10^6^ N/m^2^ can be achieved in these actuators. It appears in the first microseconds of the applied voltage pulse. The mechanical construction of the contact elements of the electrostatic micro-drives was found to be very reliable. The petals are made of highly durable material—bronze with excellent mechanical reliability that can withstand more than 1 × 10^12^ switching cycles.

After the end of the voltage pulse and the end of the rolling process, the petal returns to its initial shape in less than 1–3 µs [15,16].

## 3. Model of Energy Conversion

Let us see how the energy conversion can be used in devices that require a high driving force in short times. The electromechanical energy conversion model describes the actuator operation [12,13,14,15].

Analysis shows that at any instant *t*, the displacement of the MP *x*(*t*), the petal length *l*, its contact length *l*_r_(*t*)*,* and the gap size *d*_e_ between the MP and FP satisfy the equation:

(3) $$x=\sqrt{l^{2}-d_{e}^{2}}-\sqrt{{\left(l-l_{r}\right)}^{2}-d_{e}^{2}}-l_{r}$$

Since $d_{e}/(l-l_{r})<<1$,

(4) $$x(t)\approx \frac{d_{e}^{2}}{2(l-l_{r}(t))}-\frac{d_{e}^{2}}{2l}$$

Therefore,

(5) $$dx/dl_{r}\approx {d_{e}}^{2}/2{\left(l-l_{r}\right)}^{2}$$

In experiments, *l*_r_ was 0.5–0.8*l*. The *x*/*l* ratio can be estimated as 0.5 × 10^−2^. Therefore, the actuator stroke can be more than 1%–5% of the device length, which is higher than the similar ratio for the piezoelectric actuator, where it equals 0.1%.

The driving force *F* and energy density *A* satisfy the equation

(6) $$A\cdot b\cdot dl_{r}=F\cdot dx$$

Therefore, the maximum *F* in the initial moment is

(7) $$F=A\cdot b\cdot \frac{dl_{r}}{dx}\approx A\frac{2\cdot b\cdot {\left(l-l_{r}\right)}^{2}}{{d_{e}}^{2}}$$

The following equation can be used to describe the MP motion as a function of time, accounting for the spring compression:

(8) $$m\cdot \frac{d^{2}x}{dt^{2}}=\frac{A\cdot b\cdot {d_{e}}^{2}}{2\cdot {\left(x+{d_{e}}^{2}/2\cdot l\right)}^{2}}-F_{f}-kx$$

where *kx* is the spring force, *F*_f_ is the friction force, *F*_f_ *<< F*. After the end of the voltage pulse, the spring force acts in the direction opposite to the initial MP motion direction, thus bringing the MP to its initial position. Cyclical motion of the MP takes place.

## 4. Electrostatic Drive Operation

Numeric solutions of Equation (8) allow one to describe the main properties of the drive operation under the effect of a short voltage pulse.

Without loss of generality, let us select the following properties: the mass of MP 1.2 × 10^−2^ kg, energy density *A*_s_ = 5 J/m^2^, gap *d*_e_ = 200 µm, petal length 0.03 m, width *b =* 2 × 10^−1^ m. It is possible for several petals to act simultaneously. In this case, *b* would be the total width of these petals. The total rolling area of the drive is 6 × 10^−4^ m^2^, and the driving force is 450 N.

**Figure 2:**
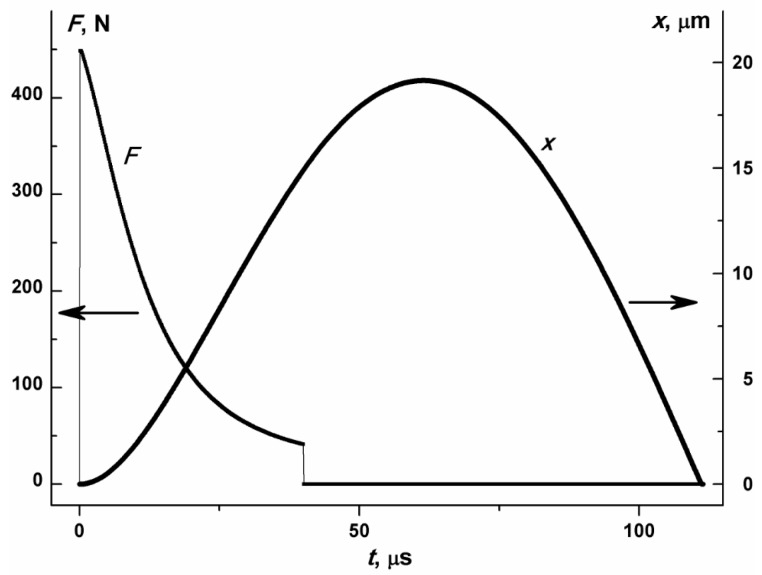
Driving force *F*, MP displacement *x*. MP mass is *m* = 1.2 × 10^−2^ kg. Voltage pulse duration *t*_p_ = 50 µs, rolling area *S = bl*_r_ = 6 × 10^−4^ m^2^, *A*_s_ = 5 J/m^2^, *d*_e_ = 200 µm.

The spring constant *k* has to be 1 × 10^7^ N/m in order to provide the full cycle time of about 100 µs with the mentioned MP mass. Figure 2 shows the driving force *F* and MP displacement *x* as functions of time during one cycle of device operation. The voltage pulse with *t*_p_ = 50 µs is applied to the device.

Figure 2 shows that a driving force of about 500 N is achieved in the first moments of the voltage pulse. This force decreases with time because the tension angle of the petal decreases. The higher the load, the more efficiently the electric field energy is used. Therefore, the pulse duration *t*_p_ has to be optimally matched with the load in order to efficiently use this energy.

Analysis of the distribution of the electric energy consumed by the drive shows that as the MP moves during the voltage pulse, this energy is transformed into the kinetic energy of the MP motion and the potential energy of the spring compression. At the same time, part of the kinetic energy is spent to compress the spring. The friction force does not noticeably affect the MP motion.

## 5. Experimental Studies

An experimental prototype of the electrostatic actuator was built to demonstrate that the described electromechanical energy converters are able to produce large mechanical forces within microsecond time frames. It was based on ceramic plates made of PbZrO_3_ with ε ~ 10000 with a 200 µm thickness. The surfaces of the ceramic plates, which are used for electrostatic rolling, are polished to optical smoothness corresponding to the 14th class of purity (the roughness is approximately 10^−8^ m). The metallic electrode (Ag film of thickness 1 µm) is applied to the other surface of the ceramic plate by vacuum deposition, followed by heating. Beryllium bronze, Cu–Be, (Be: 2%; Ni: 0.4%) with a thickness of 2–3 µm was used for the petals. These plates can be used with voltage pulses of 100–300 V, and therefore a high specific energy density can be obtained.

Our goal was to reliably determine the mechanical force that is produced by the actuator in the first 50 µs. The MP mass was 1.2 × 10^−2^ kg. The mass was glued to the MP surface because its acceleration can be as high as 5000 m/s^2^. 

**Figure 3:**
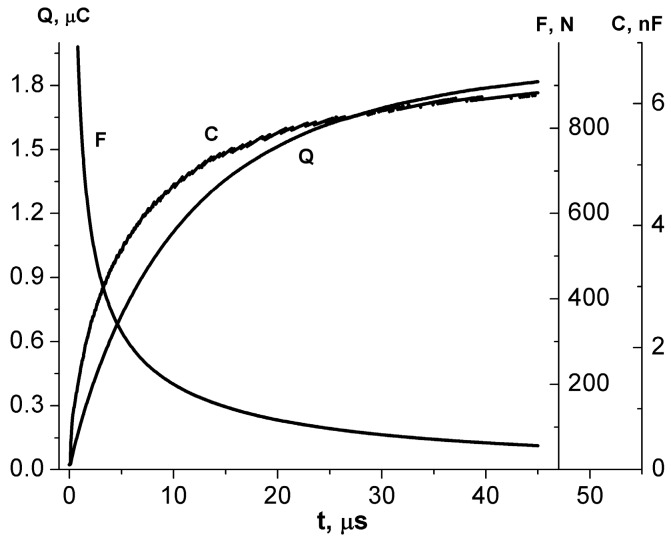
Experimental curves. Driving force (curve *F*), charge *Q* (curve *Q*) and structure capacity *C* as functions of time. Pulse voltage is 300 V, load resistance is 1 kOhm.

Direct measurement of the drive yield force *F*(*t*) in a microsecond time frame is hardly feasible. Therefore, the MP speed measurement was used to detect *F*(*t*). It is easy to estimate the force *F*(*t*) from an equation *F*(*t*) *=* (*Q*(*t*)*mV*)^0.5^/*t*, where *Q*(*t*) is the charge accumulated in the structure at time *t*. This equation follows the laws of the conservation of energy and momentum. Figure 3 shows the evolution of *Q*(*t*) corresponding to the motion of the MP with a mass of 1.2 × 10^−2^ kg in the process of electromechanical energy transformation. The curve behavior corresponds to the force *F*(*t*) and the capacitance *C*. This force is within the range 50–800 N. The MP velocity reaches 2.1 × 10^−1^ m/s by 50 μs. The motion starts within 5 μs.

**Figure 4:**
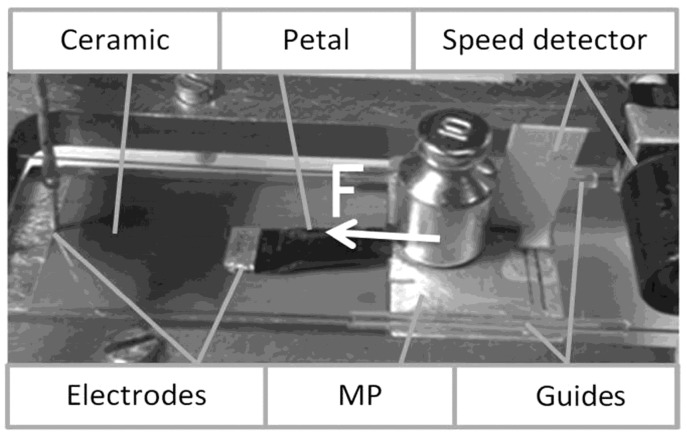
Experimental prototype of the drive, *m* = 1.2 × 10^−2^ kg, antiferroelectric ceramic PbZrO_3_, thickness *d* = 200 μm, petals beryllium bronze Cu–Be, thickness 2–3 µm, petal width is 8.4 × 10^−3^ m, its length is 1.8 × 10^−2^ m.

The experimental set is shown in Figure 4. The MP shifts along the guides. The MP motion is caused by the electrostatic rolling of a thin metal film on the ceramic surface during the voltage pulse. The gap size *d*_e_ is 3 × 10^−4^–7 × 10^−4^ m, but it can vary widely. The bronze film is located at the side of the MP for easy observation of the rolling process.

**Figure 5:**
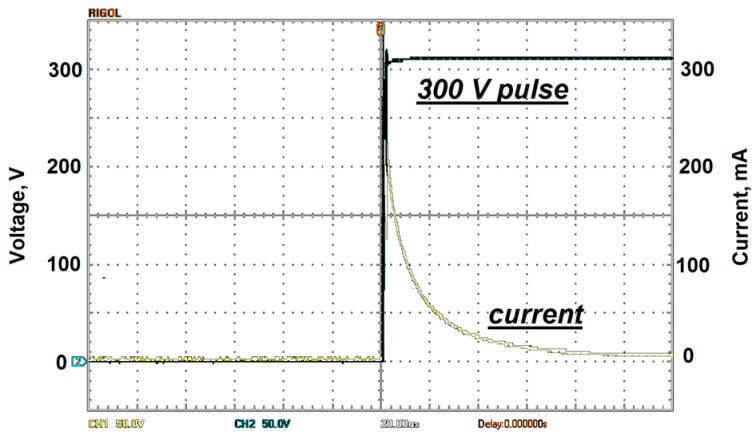
Waveforms of the 300 V voltage pulse (black) and rolling current (white); load resistance is 1 kOhm; the front edge of the pulse lasts 1 µs. Two channel digital oscilloscope DS5102CA screenshot.

Waveforms of the voltage pulse applied to the prototype and the electric current that characterizes the process of electrostatic rolling of the petal on the ceramic plate surface are presented in Figure 5.

Two channel digital oscilloscope DS5102CA (RIGOL Technologies Inc., Beaverton, OR, USA) with a 100 MHz bandwidth is used. The petal width was 8.4 × 10^−3^ m, and its length was 1.8 × 10^−2^ m. The voltage was 300 V, and the front edge of the pulse lasted 1 µs. 

The speed detector consists of a light emitter and a photo sensor couple. The MP moves between them, and a sensor signal is recorded.

The charge *Q*(*t*) formed during the electrostatic rolling process and the capacitance *C*(*t*) of the electrode–ferroelectric–gap–moving electrode structure are calculated from the waveform. 

The charge is *Q* = 1.8 × 10^−^^6^ C, *C* = 6 × 10^−^^9^ F. Thus, the average size of the gap calculated from the expression for the structure capacity is 170 nm, and *A*_s_ ~2.3 J/m^2^.

Figure 3 shows the charge *Q,* force *F* and capacity *C* as functions of time. A high driving force led to noticeable MP displacement within 5 µs. The MP displacement reached 20 µm within 50 µs. The experimental curves agree with the mathematical model of the drive with the given parameters.

The actuator construction makes it possible to considerably increase the power yield as well as its energy consumption by increasing the MP rolling area. Further, a sufficient number of petals can be placed on the surface of the MP. 

## 6. Conclusions

The analysis has shown that the described electromechanical energy conversion can be used to design high-energy-output electromechanical fast-acting drives. With the operating voltage of 100–300 V, the drive can produce mechanical forces up to 100–1000 N in the time frame of 10–50 µs, with a stroke up to 20–50 µm.

The performed analysis shows that it is possible to construct a principally new, highly efficient drive based on ferroelectric materials with a high value of the dielectric constant. Such an electrostatic actuator using a nanometer gap can be manufactured by the methods of microelectronic technology.
